# Small molecule drug discovery for glioblastoma treatment based on bioinformatics and cheminformatics approaches

**DOI:** 10.3389/fphar.2024.1389440

**Published:** 2024-04-12

**Authors:** Liya Feng, Sha Zhu, Jian Ma, Jing Huang, Xiaoyan Hou, Qian Qiu, Tingting Zhang, Meixia Wan, Juan Li

**Affiliations:** ^1^ Department of Basic Medical Sciences, College of Medicine, Longdong University, Qingyang, China; ^2^ Gansu Province Medical Genetics Center, Gansu Provincial Maternal and Child Health Hospital, Lanzhou, China; ^3^ Key Lab of Preclinical Study for New Drugs of Gansu Province, Institute of Biochemistry and Molecular Biology, School of Basic Medical Sciences, Lanzhou University, Lanzhou, China

**Keywords:** glioblastoma, multi-omics data, bioinformatics, therapeutic agents, ADMET, molecular docking

## Abstract

**Background:** Glioblastoma (GBM) is a common and highly aggressive brain tumor with a poor prognosis for patients. It is urgently needed to identify potential small molecule drugs that specifically target key genes associated with GBM development and prognosis.

**Methods:** Differentially expressed genes (DEGs) between GBM and normal tissues were obtained by data mining the Gene Expression Omnibus (GEO) and The Cancer Genome Atlas (TCGA) databases. Gene function annotation was performed to investigate the potential functions of the DEGs. A protein-protein interaction (PPI) network was constructed to explore hub genes associated with GBM. Bioinformatics analysis was used to screen the potential therapeutic and prognostic genes. Finally, potential small molecule drugs were predicted using the DGIdb database and verified using chemical informatics methods including absorption, distribution, metabolism, excretion, toxicity (ADMET), and molecular docking studies.

**Results:** A total of 429 DEGs were identified, of which 19 hub genes were obtained through PPI analysis. The hub genes were confirmed as potential therapeutic targets by functional enrichment and mRNA expression. Survival analysis and protein expression confirmed centromere protein A (CENPA) as a prognostic target in GBM. Four small molecule drugs were predicted for the treatment of GBM.

**Conclusion:** Our study suggests some promising potential therapeutic targets and small molecule drugs for the treatment of GBM, providing new ideas for further research and targeted drug development.

## Introduction

Glioblastoma (GBM) is the most common aggressive glioma with high incidence and recurrence rates (Liao et al., 2019). Its prognosis is poor, and the survival time after diagnosis is less than 15 months (Chavda et al., 2020). Currently, the clinical treatment of GBM is based on surgical resection with adjuvant radiotherapy and chemotherapy (Byrne et al., 2021). The alkylating agent temozolomide is the most widely used chemotherapy approved for the treatment of GBM, either alone or combined with radiotherapy. However, it can cause unwanted toxicity and drug resistance (Karachi et al., 2018). In addition, almost all GBM that respond to first-line therapy will relapse. The available treatment options for recurrent GBM are very limited (Tosoni et al., 2016). The DNA alkylating drugs lomustine and carmustine have been approved by the US Food and Drug Administration (FDA) for the treatment of recurrent GBM, but outcomes are also unsatisfactory (Wu et al., 2021). Therefore, the identification and analysis of potential targeted therapeutic agents for the treatment of GBM is critical.

Integrated genomics, proteomics, and bioinformatics have provided powerful new strategies for cancer drug discovery (Sharma et al., 2023). Some studies have identified small molecule drugs for GBM using multiple omics data and bioinformatics methods (Lu et al., 2020; Qi et al., 2020; Xia et al., 2022). Small molecule drugs are the preferred choice for the treatment of neurological diseases because their simple structure allows them to penetrate the central nervous system and exert their effects. Meanwhile, the cost of small molecule drugs is lower, making them more acceptable to patients. The unique advantages of small molecule drugs have made them a focus of GBM drug research (Liu H. et al., 2022).

With the advent of the Gene Expression Omnibus (GEO) and The Cancer Genome Atlas (TCGA) resources, collaborative analysis of array and sequence-based cancer data is at the forefront of drug discovery (Li et al., 2020). Previous studies have demonstrated many small molecule targets play a key role in the development of GBM and have the potential to serve as adaptable targets for the development of novel anti-GBM drugs (Mitra and Ayyannan, 2021). However, these studies have not combined the GEO and TCGA databases to perform a meta-analysis of gene expression for GBM. The molecular mechanism of GBM pathogenesis and therapies against molecular targets have not been fully elucidated. This research gap provides an opportunity to investigate new biomarkers and small molecule drugs for GBM.

This study used data mining to integrate information from the GEO and TCGA databases and identify differentially expressed genes (DEGs) in GBM. Based on these DEGs, we used a comprehensive bioinformatics approach to search for reliable biomarkers for GBM and explore potential small molecule drugs with targeted therapeutic effects. In addition, our study employed the cheminformatics approach including absorption, distribution, metabolism, excretion, toxicity (ADMET) analysis and molecular docking studies to reveal safe and effective drug-likeness molecules against GBM. Few researchers have used this computational approach to predict and identify effective small molecules against GBM. In summary, this study comprehensively used bioinformatics and chemoinformatics approaches to investigate new biomarkers and novel potential small molecule candidates to improve the therapeutic effects of GBM and provide a theoretical basis for further research.

## Material and methods

### Data collection

GBM expression profiles were retrieved from the GEO database (https://www.ncbi.nlm.nih.gov/geo/). To reduce the complexity of the analysis and ensure accurate results, eligible gene expression data were filtered based on their sample size (at least eight), appropriate conditioning (profiling both human GBM and normal tissue gene expression), no other intervention measures (no chemical or physical treatment), and proper formatting. Following these criteria, three GBM gene expression profiles (GSE137902, GSE90886, and GSE34152) met the eligibility requirements and were downloaded from the GEO repository. Furthermore, 170 GBM and five normal brain samples were selected from the TCGA data portal (https://www.cancer.gov/tcga) and used as a complementary dataset.

### Datasets analysis

After principal component analysis (PCA) was performed on the GEO datasets for dimensionality reduction and quality control, the “limma” package of R language software (version 4.3.2) (Ritchie et al., 2015) was applied to screen for the DEGs between patients with GBM and healthy controls with the criterion of |log fold change (FC)| > 2 and adjusted *p*-value <0.05. To obtain consensus on the DEGs, all data from the TCGA projects were normalized and processed using the “TCGAbiolinks” package of R software (Colaprico et al., 2016). The parameters set for the DEGs analysis were |log2 FC| >1 with adjusted *p*-value <0.05. The R language package “ggplot2” was applied to generate volcano plots to visualize the identified DEGs. We then combined the DEGs acquired from the GEO and TCGA databases to obtain common genes.

### Functional enrichment analysis

To further investigate the potential molecular mechanisms of the DEGs, Gene Ontology (GO) enrichment (Gene Ontology Consortium, 2021) and Kyoto Encyclopedia of Genes and Genomes (KEGG) annotations (Kanehisa et al., 2021) of the DEGs were performed using the “clusterProfiler” package of R software (Yu et al., 2012). Pathways with a significance level of *p* values < 0.05 were considered relevant and selected. We determined the top 10 biological processes (BP), cellular components (CC), molecular functions (MF), and signaling pathways involved in the DEGs.

### Protein-protein interaction (PPI) network construction and module analysis

Protein-protein interactions were analyzed by the STRING online database (version 12.0) (Szklarczyk et al., 2019). Based on the STRING online tool, a PPI network of the DEGs with a confidence score of at least 0.7 was constructed. Cytoscape software was then employed to visualize the PPI network using the TSV file downloaded from the STRING database. The Molecular Complex Detection (MCODE) plugin in Cytoscape software was applied to explore the significant modules in the PPI network with k-core = 2, degree cutoff = 2, max depth = 100, and node score cutoff = 0.2 (Bader and Hogue, 2003). Hub genes in the most densely connected cluster were screened and collected for functional enrichment analysis and final drug discovery.

### Gene expression and survival analysis of the hub genes

The Gene Expression Profiling Interactive Analysis (GEPIA) is a developed website containing a substantial amount of RNA sequencing expression data from the TCGA projects (Tang et al., 2017). The expression levels of the hub genes identified from the PPI network in GBM *versus* normal tissues were processed using GEPIA to further confirm their reliability. The UALCAN platform is a comprehensive web portal for analyzing cancer omics data to identify biomarkers or perform *in silico* validation of potential genes of interest (Chandrashekar et al., 2017). We performed the differential patient survival analysis in GBM on this platform to explore the prognostic significance of the hub genes and to identify key genes with significant differences in expression for further study. Additionally, we selected isocitrate dehydrogenase 1 (IDH1), a widely accepted clinical biomarker that provides prognostic or predictive information for GBM (Sareen et al., 2022), as a control gene to compare the potential of hub genes as potential therapeutic and prognostic targets.

### Validation of protein expressions of the prognostic genes

CancerSEA is the first dedicated database to analyze the different functional states of cancer cells at the single-cell level (Yuan et al., 2019). Cellular functional states include angiogenesis, apoptosis, cell cycle, differentiation, DNA damage, DNA repair, epithelial-mesenchymal transition (EMT), hypoxia, inflammation, invasion, metastasis, proliferation, quiescence, and stemness. We used the CancerSEA database to explore the functional status of prognostic genes in GBM. The protein expression of prognostic genes in GBM compared to normal tissues was investigated using immunohistochemistry (IHC) from the Human Protein Atlas (HPA) database, an online tool that allows users to analyze protein levels in clinical samples (Uhlén et al., 2015).

### Drug-gene interactions

We used DGIdb (Wagner et al., 2016), a valuable database that provides free services for searching drug-gene interaction information, to search for existing small molecule drugs based on the hub genes as potential targets. These drugs may provide new solutions for the treatment of patients with GBM. Cytoscape software was used to visualize potentially active drugs against hub genes.

### Evaluation of absorption, distribution, metabolism, excretion, and toxicity (ADMET) properties

To predict the safety and potency of small molecule drugs and to screen for drug candidates, we used ADMETlab (Xiong et al., 2021) to obtain the pharmacokinetic characteristics and toxicity profiles of the drugs. ADMETlab accepts compounds in the simplified molecular input line entry specification (SMILES) format. Potentially active drugs were screened using several parameters according to the criteria recommended by the ADMETlab server. Firstly, the ability of the compounds to cross the blood-brain barrier (BBB) was assessed. BBB values of 0.1 or higher were required for drugs to have a potential effect on the central nervous system. The drugs should have solubility values greater than −4 to ensure that they can be dissolved and effectively absorbed into the body. The distribution coefficient D had to be in the range of 1 to 5, and the distribution coefficient P had to be in the range of 0 to 3. The human intestinal absorption (HIA) values had to be equal to or greater than 0.3 to ensure the oral absorption potential of the drugs. Moreover, the human hepatotoxicity values should be less than 1 and the lethal dose (LD50) values should be 0, indicating a lower risk of drug toxicity. Finally, Lipinski’s rule of five, which is considered to determine the drug-likeness properties of small molecules, needed to be within an acceptable range.

### Molecular docking

To assess the reliability of drug treatment of GBM, molecular docking was performed between the small molecules predicted from ADMETlab and the potential target protein of GBM, and the magnitude of the binding energy was calculated. The crystal structure of the target protein was retrieved from the Protein Data Bank (PDB) database (Berman et al., 2000). We selected the target protein structures from the highly resolved ligand-protein X-ray complexes to determine the active binding site. Alternatively, we used the DeepSite platform (Jiménez et al., 2017) to detect the site of the whole protein to assess the possible area of the active binding site. The three-dimensional structures of the ligands (small molecule drugs) in SDF formats were obtained from the PubChem database (Kim et al., 2021). The Schrödinger software (version 2021) was used to perform the docking study and calculate the docking scores. The molecular docking results were visualized using PyMOL software (version 2.4.1) (Seeliger and de Groot, 2010).

## Results

### Identification of DEGs

**FIGURE 1:**
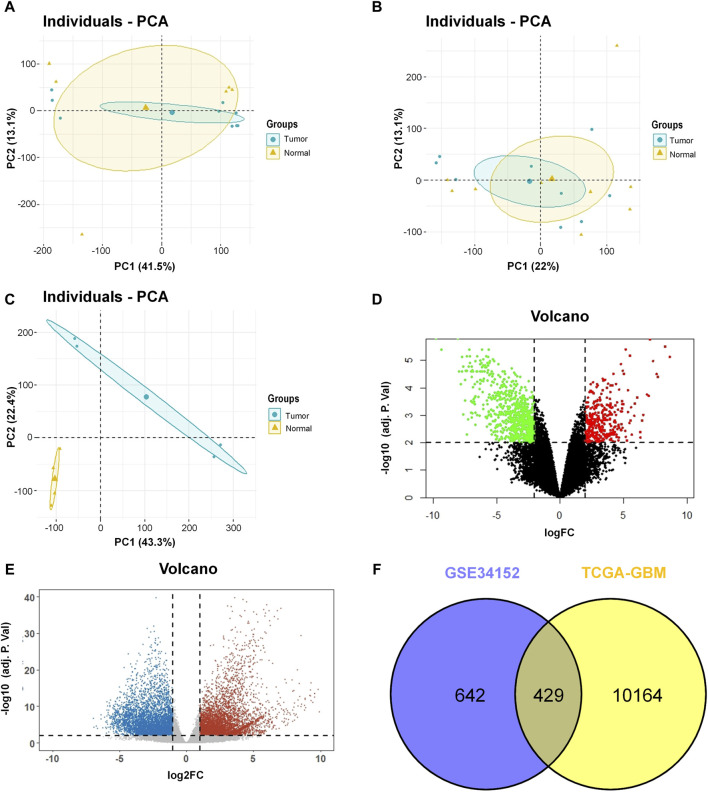
Identification of DEGs in the GEO database and the TCGA project. **(A)** Result of PCA for GSE137902. **(B)** Result of PCA for GSE90886. **(C)** Result of PCA for GSE34152. **(D)** Volcano plot of the DEGs in GSE34152. **(E)** Volcano plot of the DEGs in TCGA. **(F)** Venn diagram of DEGs between the GEO database and the TCGA project. DEGs, differentially expressed genes; GEO, Gene Expression Omnibus; TCGA, The Cancer Genome Atlas; PCA, principal component analysis.

Gene expression profiling and sequencing data of GBM were downloaded from the GEO and TCGA databases. Detailed information on the datasets is provided in Table 1. To distinguish the significant difference between the normal and tumor samples of the GEO datasets, PCA was performed to reduce the dimensionality and evaluate the independence of each group. The results showed that tumor samples in the GSE137902 and GSE90886 datasets were close to the normal samples (Figures 1A,B), whereas, the normal and tumor samples in the GSE34152 dataset displayed a significant difference (Figure 1C). Therefore, we retained the GSE34152 dataset for subsequent analysis. The volcano plots in Figure 1D showed 1,586 DEGs screened out from the GSE34152 dataset, including 560 upregulated genes and 1,026 downregulated genes. For the TCGA-GBM data, we found a total of 10593 DEGs with 5,503 upregulated and 5,090 downregulated genes (Figure 1E). The Venn diagram demonstrates the intersection of genes between the GEO and TCGA data, and 429 common DEGs were found (Figure 1F). These 429 genes were further subjected to functional annotation and protein-protein interaction analysis to determine the biological significance of this cross-study convergence in GBM pathogenesis.

**TABLE 1:** Characteristics of datasets in this study.

Dataset	Platform	Sample		Total sample	Publication
		Normal	Glioblastoma		
GSE137902	GPL13667	6	9	15	Nature Communications
GSE90886	GPL15207	9	9	18	Biomed Research International
GSE34152	GPL570	4	4	8	Plos One
TCGA-GBM	-	5	170	175	-

### GO and KEGG enrichment analysis of DEGs

**FIGURE 2:**
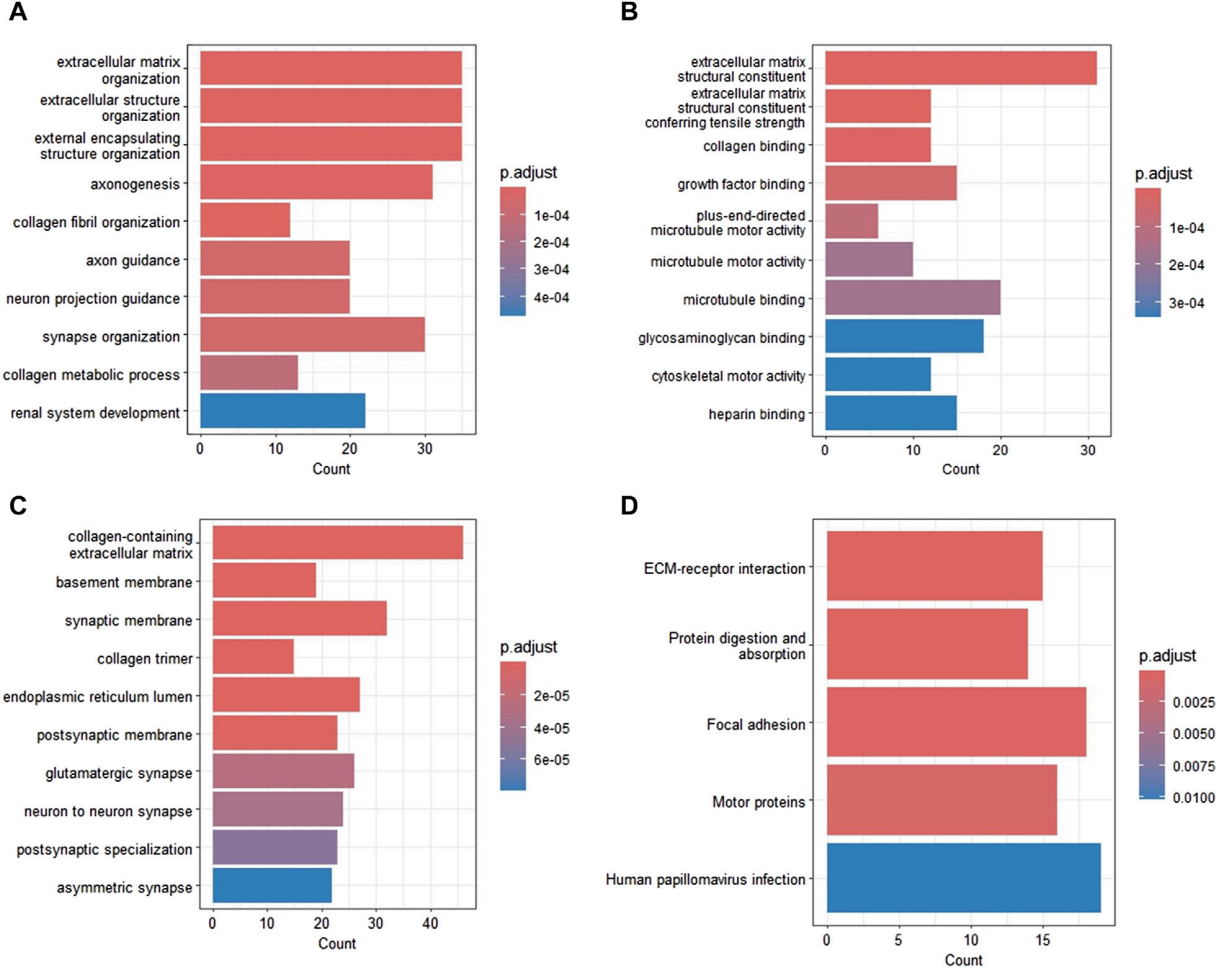
Functional enrichment analysis of DEGs in GBM. **(A)** Biological processes. **(B)** Molecular function. **(C)** Cellular components. **(D)** KEGG pathways. DEG, differentially expressed gene; GBM, glioblastoma; KEGG, Kyoto Encyclopedia of Genes and Genomes.

GO and KEGG functional enrichment analyses were performed to further elucidate the potential mechanisms of DEGs. The most enriched GO_BP annotations included: extracellular matrix (ECM) organization, extracellular structure organization, and external encapsulating structure organization (Figure 2A). GO_MF annotations were significantly enriched in ECM structural constituent, ECM structural constituent conferring tensile strength, and collagen binding (Figure 2B). The most enriched GO_CC categories were collagen-containing ECM, basement membrane, and synaptic membrane (Figure 2C). Figure 2D shows the most prominent pathways in the KEGG pathway analysis. The significantly enriched pathways were ECM-receptor interaction, protein digestion and absorption, focal adhesion, and the motor protein.

### PPI network analysis of DEGs

**FIGURE 3:**
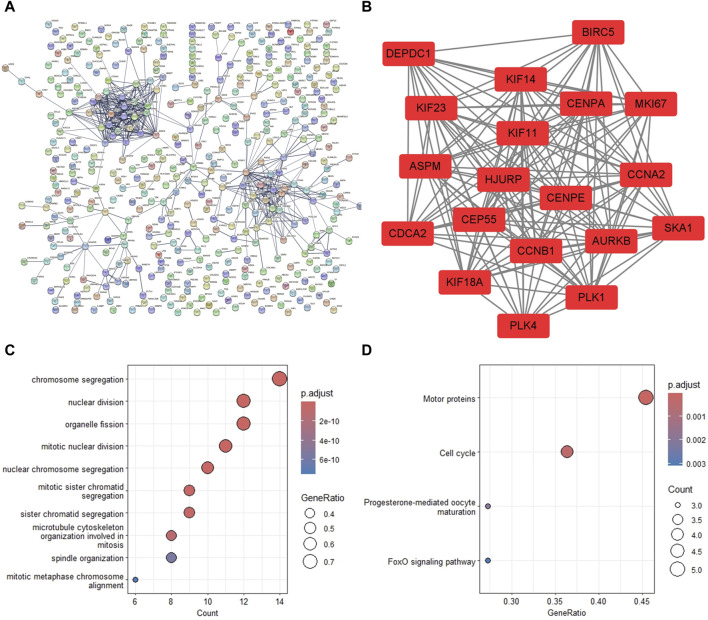
PPI network construction and significant gene module analysis. **(A)** The PPI network of the common DEGs. **(B)** The most significant gene module extracted from the PPI network. **(C)** The biological processes of analysis of the module genes. **(D)** The KEGG pathway analysis of the module genes. DEG, differentially expressed gene; PPI, protein-protein interaction; KEGG, Kyoto Encyclopedia of Genes and Genomes.

To investigate the association of the DEGs, the PPI network was constructed with 417 nodes and 540 edges, where nodes represented genes, and edges represented connections between two genes (Figure 3A). The most significant module was extracted from the PPI network using MCODE arithmetic (Figure 3B). According to this screening method, we obtained 19 hub genes of DEGs. The 19 genes included kinesin family member 23 (KIF23), abnormal spindle microtubule assembly (ASPM), centromere protein A (CENPA), aurora kinase B (AURKB), DEP domain containing 1 (DEPDC1), the marker of proliferation Ki-67 (MKI67), baculoviral IAP repeat containing 5 (BIRC5), centrosomal protein 55 kDa (CEP55), centromere protein E (CENPE), cyclin A2 (CCNA2), holliday junction recognition protein (HJURP), kinesin family member 11 (KIF11), cyclin B1 (CCNB1), kinesin family member 18A (KIF18A), polo-like kinase 1(PLK1), polo-like kinase 4 (PLK4), cell division cycle associated 2 (CDCA2), spindle, and kinetochore associated complex subunit 1(SKA1), and kinesin family member 14 (KIF14). The most significant enrichment pathways of 19 hub genes in GO_BP and KEGG terms were the chromosome segregation pathway (Figure 3C) and the motor protein pathway (Figure 3D), respectively.

### Validation of the hub genes

**FIGURE 4:**
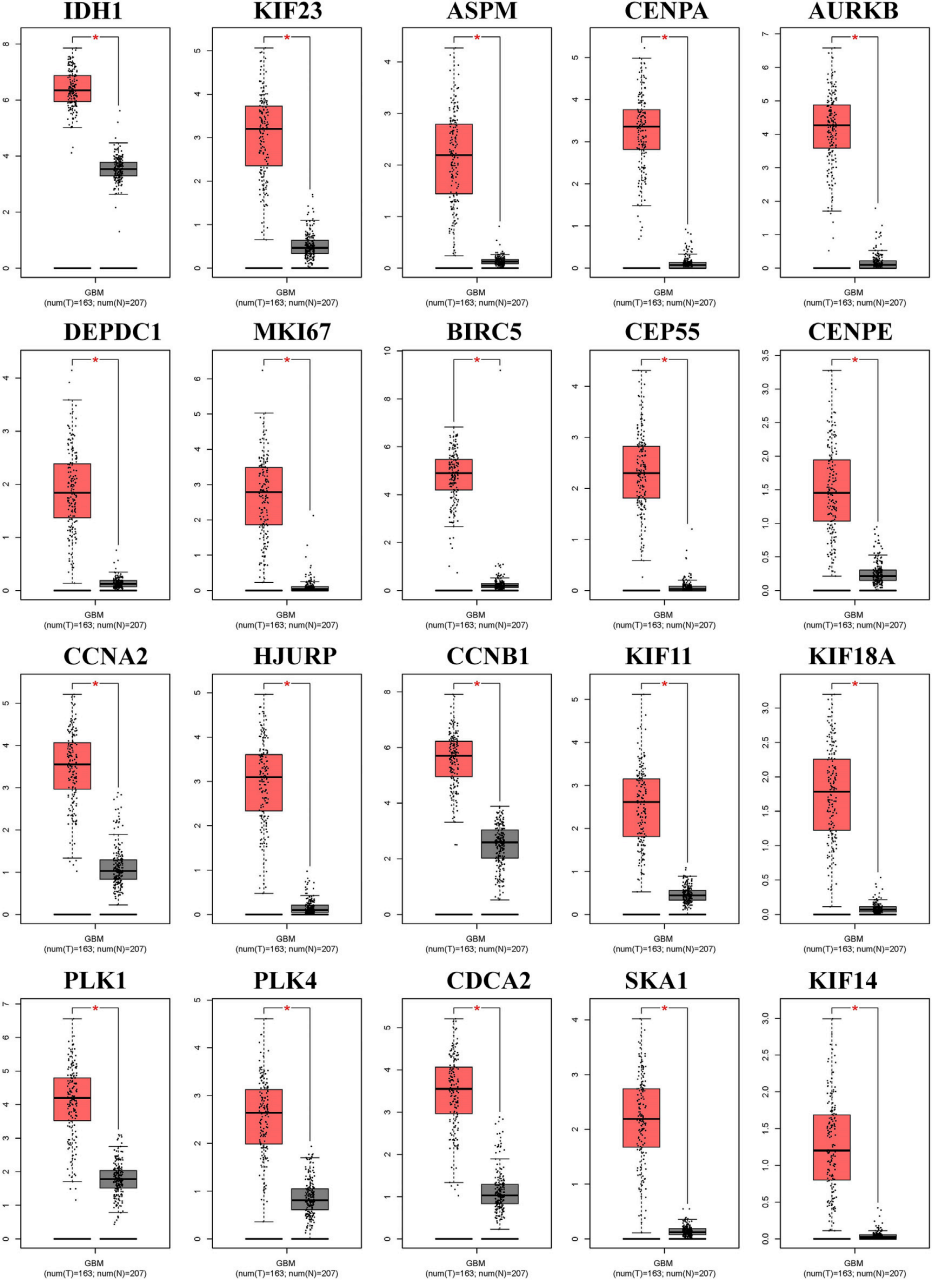
Expression levels of IDH1 and 19 hub genes between the normal and tumor groups in GBM. IDH1, isocitrate dehydrogenase 1; GBM, glioblastoma.

**FIGURE 5:**
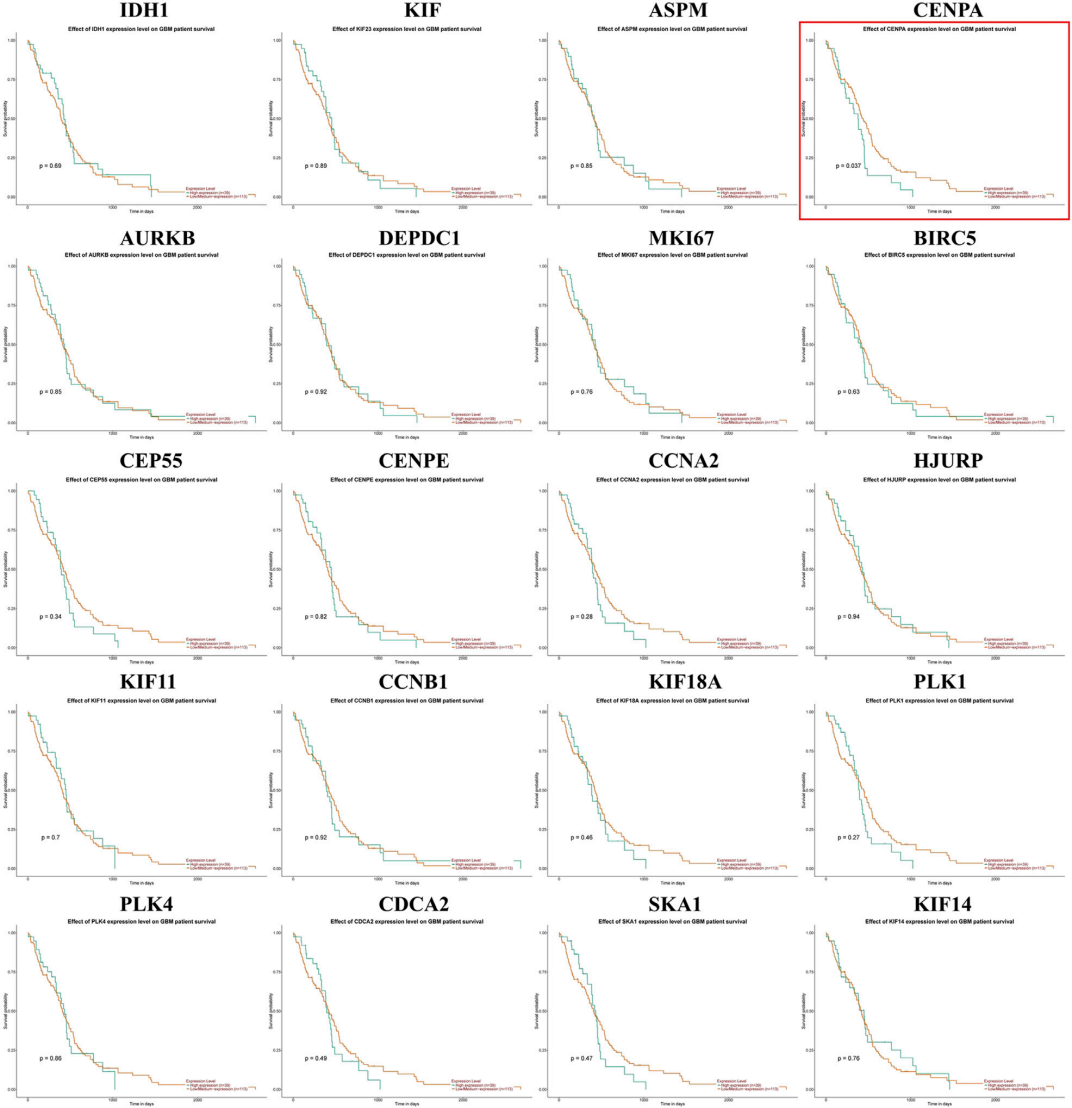
Effect of IDH1 and hub gene expression levels on survival of GBM patients. IDH1, isocitrate dehydrogenase 1; GBM, glioblastoma.

**FIGURE 6:**
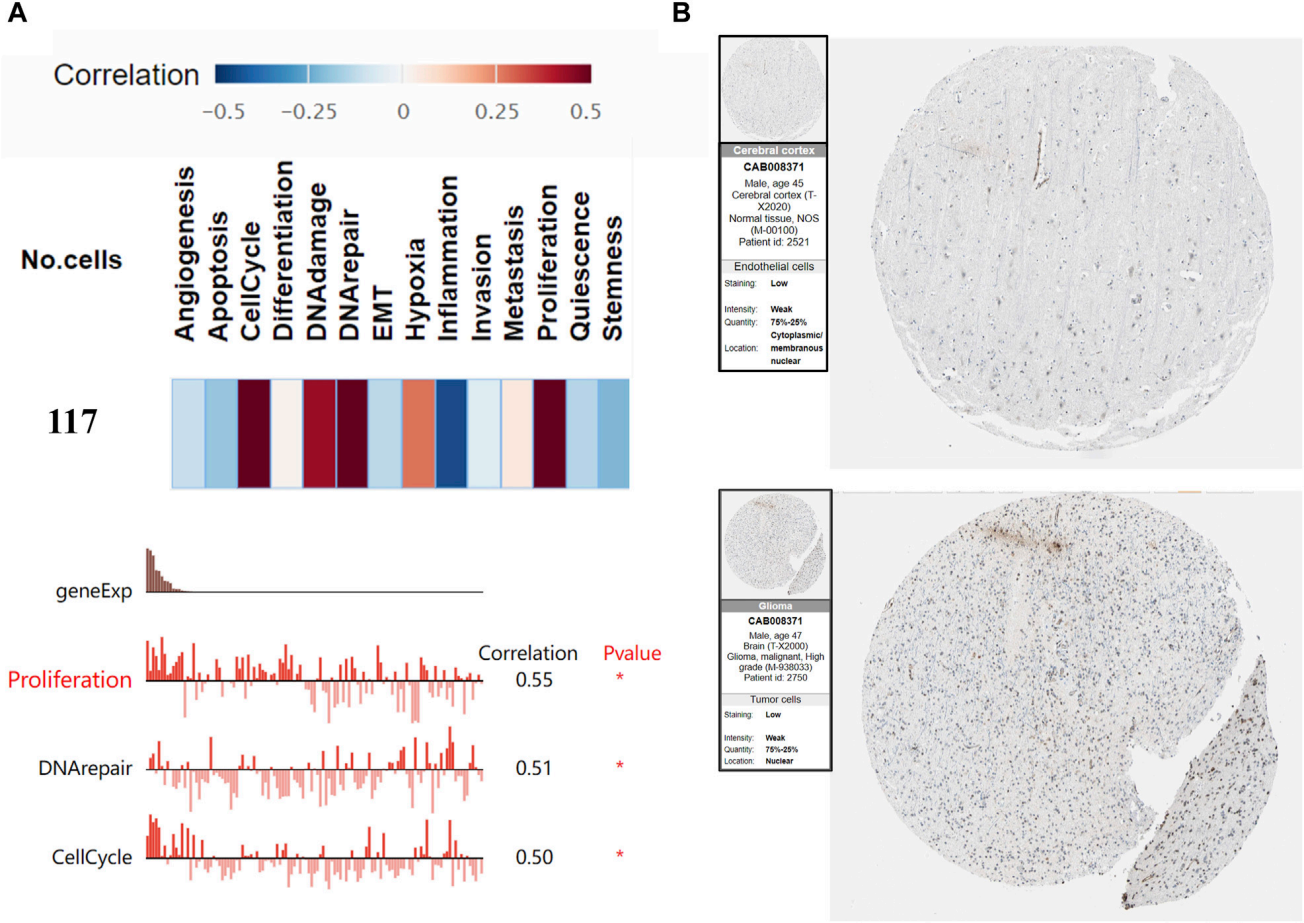
Functional relevance and protein levels of CENPA in GBM. **(A)** Correlations between CENPA and functional states in GBM. **(B)** Protein expression of CENPA in immunohistochemical images of GBM and normal tissue. GBM, glioblastoma.

We generated the differential expression level analysis of the 19 hub genes and the control gene IDH1 using GEPIA. The results are presented as box plots (Figure 4). Similar to IDH1, the expression level of hub genes was significantly upregulated in GBM compared to normal samples. The results confirmed that the expression levels of these hub genes were closely related to GBM onset. Thus, we selected all hub genes for drug-gene interaction analysis. To explore the potential prognostic value of the hub genes and IDH1, survival curves were generated on the UALCAN platform (Figure 5). Among the 19 hub genes, the expression level of CENPA correlated significantly with the survival of GBM patients (*p*-value <0.05). In contrast, the expression level of IDH1 was not significantly correlated with survival in GBM patients (*p*-value >0.05). As shown in Figure 4, we also observed the high expression of CENPA in GBM samples compared to that in normal samples. Moreover, to explore how CENPA might affect cancer pathogenesis, we used the CancerSEA single-cell database to analyze the correlation between CENPA and 14 distinct functions. Functional relevance analysis showed that CENPA expression was positively correlated with proliferation, DNA repair, and cell cycle in GBM (Figure 6A). The IHC results from the HPA database suggested that the protein level of CENPA was lower in normal samples, whereas the level was high in GBM samples (Figure 6B). Thus, CENPA may serve as a potential biomarker for GBM diagnosis and prognosis.

### Searching for small molecule therapeutic drugs

**FIGURE 7:**
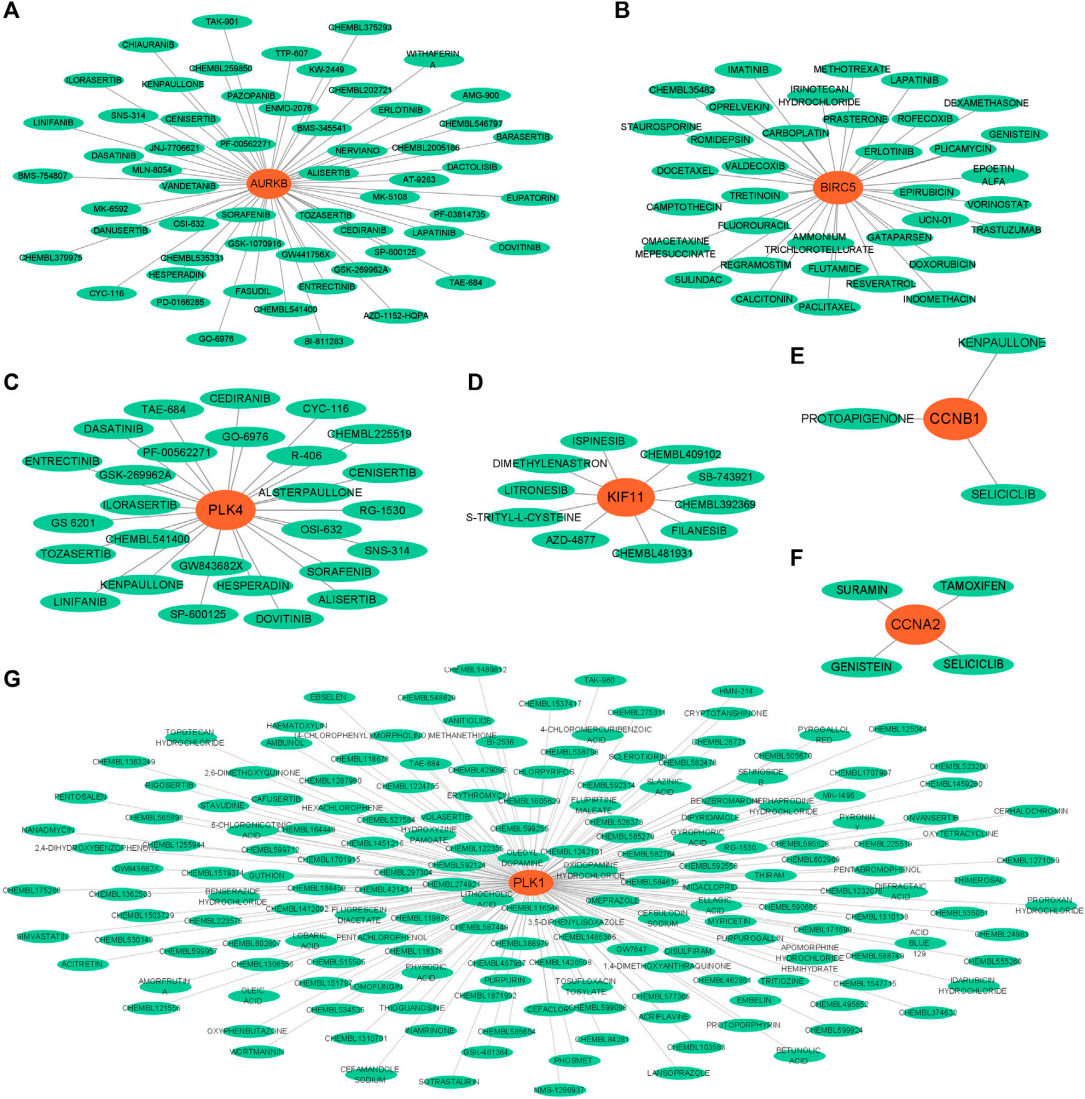
Gene-drug interaction networks constructed with hub genes and small molecule drugs. **(A)** AURKB. **(B)** BIRC5. **(C)** PLK4. **(D)** KIF11. **(E)** CCNB1. **(F)** CCNA2. **(G)** PLK1.

Through querying the DGIdb database, we explore the interaction between the hub genes and available therapeutic drugs for cancer. The hub gene-drug interaction network was visualized using Cytoscape (Figure 7). We found that seven out of 19 genes could correspond to specific small molecule drugs. The potential drug target genes were AURKB (58 drugs), BIRC5 (36 drugs), PLK4 (27 drugs), KIF11(10 drugs), CCNB1 (three drugs), CCNA2 (four drugs), and PLK1 (176 drugs). After removing duplicate names of the same drugs targeting different genes, 284 drugs were selected as possible drugs for GBM treatment.

### Cheminformatics prediction for drugs

We carried out the initial virtual screening of 284 small molecules by assembling the pharmacokinetic properties and toxicity profiles of drugs using the ADMETlab web server. As observed in Table 2, the four compounds exhibited excellent ADMET properties. The selected compounds were predicted to be able to cross the BBB. The aqueous solubility values of the selected compounds were within the recommended range. We found the compounds had high absorption levels in the HIA. Our analysis also provided human hepatotoxicity and LD50 values for the four molecules, which were predicted to be safe and non-toxic for administration. Additionally, the final compounds were predicted to have good bioavailability and to meet Lipinski’s rule of five drug-likeliness parameters. The chemical structures of these four compounds are listed in Table 3.

**TABLE 2:** ADMET characteristics of the top ranked drugs.

Small molecule drugs	Solubility	Distribution coefficient D	Distribution coefficient P	Human intestinal absorption	Blood-brain barrier	Human hepatotoxicity	LD50_oral	Lipinski
AT-9283	−3.987	2.567	1.607	0.986	0.754	0.722	0	Accepted
Seliciclib	−2.687	2.915	2.84	0.534	0.546	0.993	0	Accepted
Litronesib	−3.634	2.212	2.403	0.652	0.944	0.375	0	Accepted
CHEMBL1310138	−2.808	1.763	1.316	0.345	0.138	0.422	0	Accepted

**TABLE 3:** Chemical structure information of the top ranked drugs.

Small molecule drugs	SMILES	2D structure
AT-9283	O=C(Nc1c[nH]nc1-c1nc2ccc(CN3CCOCC3)cc2[nH]1)NC1CC1	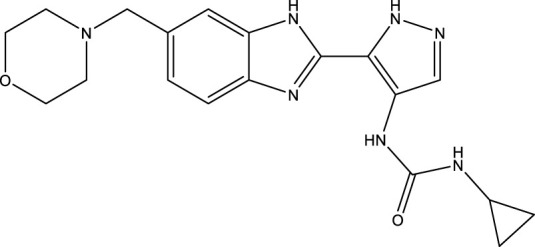
Seliciclib	CC[C@H](CO)Nc1nc(NCc2ccccc2)c2ncn(C(C)C)c2n1	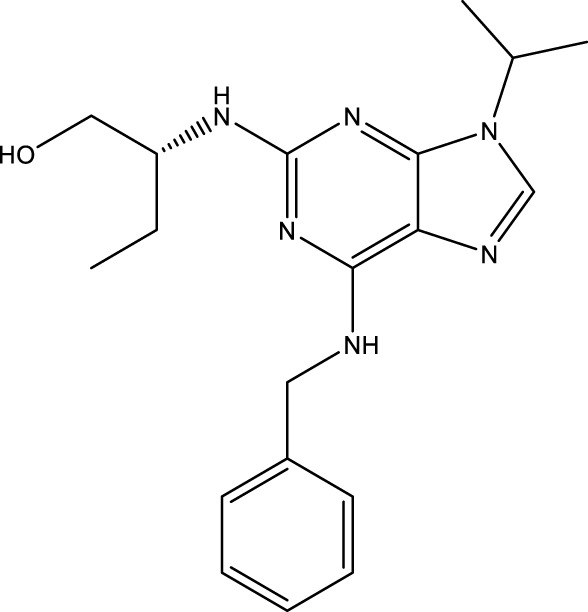
Litronesib	CCNCCS(=O)(=O)NC[C@@]1(c2ccccc2)SC(NC(=O)C(C)(C)C) = NN1C(=O)C(C)(C)C	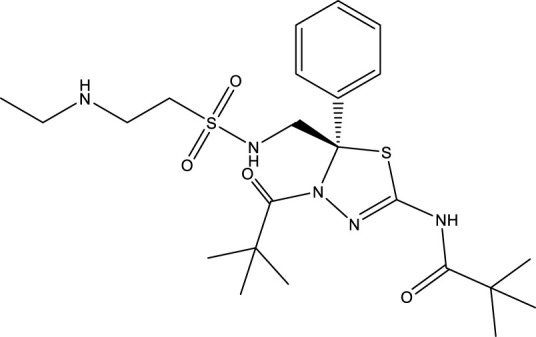
CHEMBL1310138	CSc1nc(S)c2[nH]c (S)nc2n1	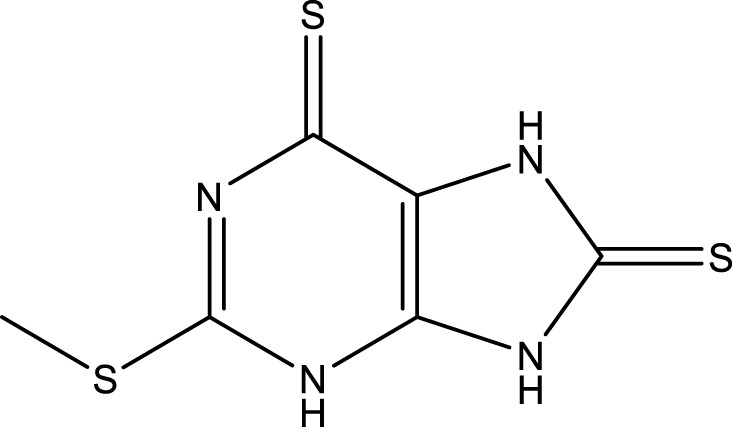

**FIGURE 8:**
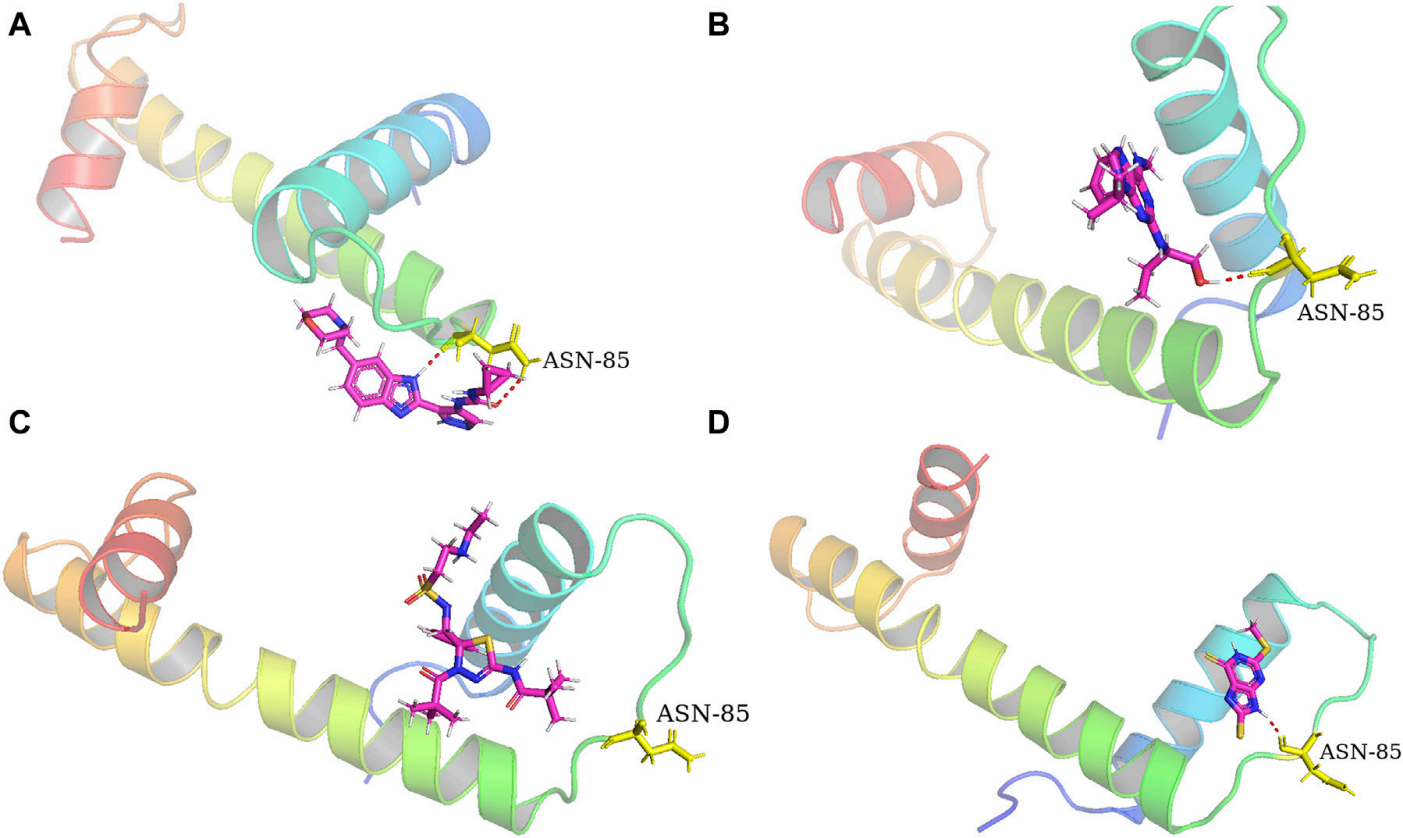
Docking diagram of the top ranked small molecule drugs with CENPA. **(A)** AT-9283. **(B)** Seliciclib. **(C)** Litronesib. **(D)** CHEMBL1310138.

Molecular docking assays with the optimized ligand structures and the protein structure of the hub genes were used to further validate the binding affinities between the four drugs and the potential biomarkers. The X-ray crystal structures of ASPM, KIF14, and DEPAC1 were not available and therefore could not be docked. The target protein binding sites of CENPA, HJURP, CDCA2 and SKA1 were estimated on the DeepSite platform. As shown in Table 4, the binding energies of the docking results were very stable, with values ranging from −2.3 to −8.5 kcal/mol. Small molecule drugs docked to the prognostic gene CANPA target were selected for docking visualization (Figure 8). The dotted lines in the figure represent hydrogen bonds. AT-9283 exhibits the strong binding affinity with CENPA (−3.981 kcal/mol) due to the formation of two hydrogen bonds with ASN 85. The interaction analysis also shows the high binding affinity of seliciclib (−3.834 kcal/mol) and litronesib (−3.592 kcal/mol) with CENPA (Figures 8B,C). Figure 8D shows that CHEMBL 1310138 interacts most strongly with CENPA (−5.084 kcal/mol). The molecular docking results indicated that the drug candidates had a potential as targeted therapies for GBM.

**TABLE 4:** Binding affinities of small molecules to target proteins.

Gene name	Protein ID	PDB ID	Docking score (kcal/mol)			
			AT-9283	Seliciclib	Litronesib	CHEMBL 1310138
KIF23	Q02241	3VHX	−5.347	−3.584	−2.318	−4.634
CENPA	P49450	3NQU	−3.981	−3.834	−3.592	−5.084
AURKB	Q96GD4	4AF3	−6.552	−6.284	−3.756	−6.477
MKI67	P46013	5J28	−2.705	−3.481	−2.487	−4.162
BIRC5	O15392	7LBK	−3.972	−3.613	−4.230	−3.891
CEP55	Q53EZ4	3WUT	−3.729	−4.373	−2.945	−4.283
CENPE	Q02224	6M4I	−4.808	−5.158	−3.096	−4.197
CCNA2	P20248	4FX3	−4.515	−5.011	−4.332	−4.776
HJURP	Q8NCD3	3R45	−4.459	−3.485	−4.203	−4.898
KIF11	P52732	6G6Z	−6.264	−7.231	−8.467	−5.669
CCNB1	P14635	4YC3	−5.487	−4.515	−2.921	−5.075
KIF18A	Q8NI77	3LRE	−4.492	−3.848	−3.604	−5.331
PLK1	P53350	5TA8	−7.301	−8.356	−3.294	−7.119
PLK4	O00444	4YUR	−6.562	−6.263	−5.784	−5.609
CDCA2	Q69YH5	5IOH	−4.242	−3.087	−2.933	−4.806
SKA1	Q96BD8	4CA0	−5.506	−4.687	−2.652	−4.708

## Discussion

GBM is a particularly aggressive and malignant type of brain tumor known for its high recurrence and low survival rates. Pharmacological treatment of GBM remains a challenge due to increasing resistance to marketed drugs (Cha et al., 2020). Therefore, it is imperative to develop novel and potent therapeutic drugs for GBM treatment. In the last decade, the explosion of omics data has provided an opportunity for computational prediction of anti-cancer drugs, improving the efficiency of drug discovery. High-throughput transcriptome data have been widely used for biomarker identification and drug prediction by integrating drug-cheminformatics data (Raslan et al., 2023). This study aimed to identify novel biomarkers and targeted small molecule drugs for GBM treatment using bioinformatics and cheminformatics methods based on multi-omics data.

We performed an integrated analysis of multiple arrays and identified 429 intersections of DEGs between the GEO and TCGA data. GO and KEGG analyses indicated that DEGs were significantly associated with ECM organization, ECM structural constituent, collagen-containing ECM, and ECM-receptor interaction pathway. Increasing evidence indicates that ECM is an essential component of the tumor microenvironment during tumor development and progression (Lee et al., 2021). The interaction between GBM cells and the tumor microenvironment promotes tumor infiltration into healthy brain tissue (Erices et al., 2023). These results are consistent with the existing research findings on GBM and reflect the close correlation between the DEGs and GBM.

We further selected 19 hub genes based on topological assessments from the PPI network of DEGs. Functional enrichment analyses were conducted to investigate the biological function of the hub genes. We found that the hub genes are primarily involved in chromosome segregation and motor protein pathways, which are closely associated with the development of GBM (de Almeida Magalhães et al., 2020; Lim et al., 2023). The hub genes were also confirmed to be overexpressed in GBM tissues compared to normal tissues, as was the accepted biomarker IDH1. Thus, these hub genes were thought to be the primary drivers of the molecular process of GBM and the underlying biomarkers for GBM therapies. Compared to previous findings, the hub genes identified in this study were not exactly consistent with their results (Shergalis et al., 2018; Alshabi et al., 2019). Part of the reason for the differences may be that our studies used different datasets and analysis methods.

According to the survival data, over-expression of CENPA was significantly associated with poorer survival in patients with GBM, more so than the known prognostic marker IDH1. Considering the above findings, our study identified CENPA as a GBM biomarker that may be a crucial and essential target for prognosis and therapy. CENPA, a histone H3 variant found in the centromeric chromatin, is critical for chromosome segregation and the maintenance of genome integrity through cell division. Importantly, CENPA overexpression has been identified in many cancers (Renaud-Pageot et al., 2022). It has been shown that CENPA could interfere with the normal progression of mitosis and regulate the tumor immune microenvironment favoring glioma development. Its expression level is significantly correlated with glioma grade (Wang et al., 2022). Previous studies have reported that CENPA is associated with the prognosis of GBM and may be a potential therapeutic strategy for GBM (Chen et al., 2020). To date, no studies have yet reported a role for CENPA in the initiation or progression of GBM. However, we found that CENPA was positively associated with various functions in GBM. CENPA may regulate cancer by mediating proliferation, DNA repair, and the cycle of GBM cells. In addition, the protein expression of CENPA was significantly higher in GBM tissues than in normal tissues as detected by IHC. Our studies provide additional evidence for the prognostic and therapeutic value of CENPA in GBM.

Repurposing old drugs as new inhibitors for cancer treatment has become an important strategy for the development of anti-tumor drugs (Yang et al., 2021). We used the DGIdb database to identify potential small molecule drugs highly related to hub genes for GBM treatment. Most of these small molecules are gene inhibitors. The pharmacokinetic properties and toxicity profiles of small molecules for oral administration were calculated in SMILES formats using ADMETlab to evaluate and screen the final drug candidates. The treatment of GBM is a predominant challenge in chemotherapy due to the existence of the BBB, which restricts the delivery of chemotherapeutic agents to the brain (Choudhury et al., 2018). Our results showed that four small molecules, including AT-9283, seliciclib, litronesib and CHEMBL 1310138, may have the ability to cross the BBB, while the water solubility and lipophilicity values of the selected compounds were within the recommended range. We also found these compounds had good absorption distribution properties in HIA. Combined with the toxicity profiles of the drug compounds and Lipinski’s rule of five, these four compounds were predicted to be the most promising drug candidates.

The final small molecules selected had potential anti-tumor activity. AT-9283, a small molecule multi-targeted kinase inhibitor, has potential as a cancer treatment due to its ability to inhibit the growth and survival of tumor cells (Torrente et al., 2020). Seliciclib, a broad cyclin-dependent kinase inhibitor, plays a potential role in cancer therapy and has undergone drug development and clinical testing as an anti-cancer agent (He and Lin, 2021). Litronesib (LY2523355) is an allosteric inhibitor of Eg5, a mitotic kinesin motor protein overexpressed in many cancers. Litronesib shows potent anti-tumor activity by inducing mitotic arrest and apoptosis in cancer cells (Garcia-Saez and Skoufias, 2021). CHEMBL 1310138 (NSC19063), a purine derivative, is also an inhibitor with apparent selectivity for Eg5 (Bosc et al., 2019).

Molecular docking verification were further explored the possibility of small molecules to treat GBM. A binding energy less than 0 indicates spontaneous binding of the ligand and receptor. The lower the binding energy, the more stable the binding conformation and the greater the likelihood of action (Liu Y. et al., 2022). The four small molecules had strong binding interactions with the target proteins, indicating that the target protein has a good affinity for the drug and that small molecule drugs are likely to act on these targets. To validate the four compounds as drug candidates to improve the prognosis of GBM, we analyzed the interaction between CENPA and small molecules. The compounds showed good docking scores with CENPA, leading to the formation of stable complexes. The binding affinities between the ligands and the target protein suggested that the drug candidates could affect the expression of CENPA. However, the potential of these molecules as drug candidates for GBM needs to be further investigated through molecular dynamics simulations and experimental supports including pre-clinical and prospective clinical studies.

Although this study contributed to the search and preliminary verification of the potential biomarkers and small drug candidates in GBM based on integrated transcriptomics, bioinformatics, and cheminformatics approaches, there were some limitations to our study. The main limitation of this study is the lack of experimental validation *in vivo* and *in vitro*, which we plan to address in future research. In addition, the limitations of this study are related to the limited data sources of the databases used. Our analysis may need to be repeated as the databases become more comprehensive. Therefore, the results of this study should only be considered as a primary prediction, which may be subject to slight changes with further experimentation.

## Conclusion

Through integrated bioinformatic analysis of GBM-related gene expression profiles from the GEO database and the TCGA project, we identified common DEGs. Functional annotations and KEGG pathways clearly illustrated the biological and pathogenic processes of GBM. Our study also revealed 19 hub genes that play important roles in disease treatment and further validated CENPA as a potential biomarker for GBM prognosis. The results of the cheminformatics analyses predicted that four potential small molecules may be safe and effective for the treatment of GBM. To the best of our knowledge, this is the first study to combine the GEO and TCGA databases to perform a comprehensive analysis of gene expression for GBM and to use cheminformatics for drug screening. Although these findings need to be verified through further molecular dynamics simulations, and *in vitro* and *in vivo* biochemical and clinical experiments, our studies still provide strong evidence to guide future research into GBM therapies.

## Data Availability

The original contributions presented in the study are included in the article/Supplementary material, further inquiries can be directed to the corresponding author.

## Abbreviations

GBM, Glioblastoma; FDA, the US Food and Drug Administration; GEO, Gene Expression Omnibus; TCGA, The Cancer Genome Atlas; PCA, principal component analysis; DEGs, differentially expressed genes; GO, Gene Ontology Consortium; KEGG, Kyoto Encyclopedia of Genes and Genomes; BP, biological processes; MF, molecular functions; CC, cellular component; PPI, protein-protein interaction; MCODE, Molecular Complex Detection; GEPIA, Gene Expression Profiling Interactive Analysis; EMT, epithelial-mesenchymal transition; HPA, Human Protein Atlas; IHC, immunohistochemistry; ADMET, absorption, distribution, metabolism, excretion, and toxicity; SMILES, simplified molecular input line entry specification; HIA, human intestinal absorption; BBB, blood-brain barrier; LD50, lethal dosage; PDB, Protein Data Bank; ECM, extracellular matrix; IDH1, isocitrate dehydrogenase 1; KIF23, kinesin family member 23; ASPM, abnormal spindle microtubule assembly; CENPA, centromere protein A; AURKB, aurora kinase B; DEPDC1, DEP domain containing 1; MKI67, the marker of proliferation Ki-67; BIRC5, baculoviral IAP repeat containing 5; CEP55, centrosomal protein 55 kDa; CENPE, centromere protein E; CCNA2, cyclin A2; HJURP, holliday junction recognition protein; KIF11, kinesin family member 11; CCNB1, cyclin B1; KIF18A, kinesin family member 18A; PLK1, polo-like kinase 1; PLK4, polo-like kinase 4; CDCA2, cell division cycle associated 2; SKA1, spindle, and kinetochore associated complex subunit 1; KIF14, kinesin family member 14.
